# AMST^2^: aggregated multi-level spatial and temporal context-based transformer for robust aerial tracking

**DOI:** 10.1038/s41598-023-36131-2

**Published:** 2023-06-04

**Authors:** Hasil Park, Injae Lee, Dasol Jeong, Joonki Paik

**Affiliations:** 1 Department of Image, Chung-Ang University, 84 Heukseok-ro, Seoul, 06974 Korea; 2 Department of Artificial Intelligence, Chung-Ang University, 84 Heukseok-ro, Seoul, 06974 Korea

**Keywords:** Applied mathematics, Computer science

## Abstract

Recently, many existing visual trackers have made significant progress by incorporating either spatial information from multi-level convolution layers or temporal information for tracking. However, the complementary advantages of both spatial and temporal information cannot be leveraged when these two types of information are used separately. In this paper, we present a new approach for robust visual tracking using a transformer-based model that incorporates both spatial and temporal context information at multiple levels. To integrate the refined similarity maps through multi-level spatial and temporal encoders, we propose an aggregation encoder. Consequently, the output of the proposed aggregation encoder contains useful features that integrate the global contexts of multi-level spatial and the temporal contexts. The feature we propose offers a contrasting yet complementary representation of multi-level spatial and temporal contexts. This characteristic is particularly beneficial in complex aerial scenarios, where tracking failures can occur due to occlusion, motion blur, small objects, and scale variations. Also, our tracker utilizes a light-weight network backbone, ensuring fast and effective object tracking in aerial datasets. Additionally, the proposed architecture can achieve more robust object tracking against significant variations by updating the features of the latest object while retaining the initial template information. Extensive experiments on seven challenging short-term and long-term aerial tracking benchmarks have demonstrated that the proposed tracker outperforms state-of-the-art tracking methods in terms of both real-time processing speed and performance.

## Introduction

Visual tracking of an object of interest is a highly important and challenging research topic in computer vision^1^. The main objective of visual tracking is to estimate the location and size of an arbitrary object in a sequence of video frames by establishing correspondences between similar pixels in different frames. In recent years, with the growing importance and usage of unmanned aerial vehicles (UAVs) such as drones, various visual tracking methods that use aerial data have been studied^2,3^. Despite considerable advances in visual tracking, aerial tracking still faces the numerous challenges, including real-time tracking, illumination fluctuation, occlusion, rapid movement, background clutter, and blurring.

Conventional visual tracking paradigms can be categorized into two categories: (1) tracking-by-detection and (2) Siamese network-based tracking.

Tracking-by-detection method first detects the object in each video frame, and then updates the object’s location using a motion model. The discriminate correlation filter (DCF) is a representative tracking-by-detection method, which uses Fourier transforms to efficiently compute cross correlation computation and achieves real-time processing^4–11^. The DCF tracker also employs hand-crafted features such as histogram of oriented gradients (HOG) to represent the object and the background. However, the DCF tracker suffers from some limitations such as the inability to handle scale changes and significant appearance variations.

Using the deep features of convolutional neural networks (CNNs), deep learning-based methods have made greater advancements in tracking performance than DCF-based trackers^12–18^. Despite advances in deep learning-based trackers, some algorithms lack computational resources that make them unsuitable for embedded platforms, while others cannot deliver the desired level of tracking performance. Until recently, DCF-based trackers were frequently employed in low-end applications, ignoring their weaker tracking performances compared to deep learning-based methods due to device constraints like those in embedded platforms.

Recently, many trackers have adopted the Siamese network architecture to simultaneously achieve both real-time processing and high performance. Siamese network-based trackers estimate an object’s position using a similarity map generated from the target appearance of a template frame and a corresponding feature representation of a search region within the search frame. These trackers are trained offline on a large dataset, such as ImageNet^19^, to measure the similarity between template and search patches. Although the original version of the Siamese tracker is SINT^20^, the most popular method is called SiamFC^21^, which has contributed to many other trackers^22–35^. Several Siamese trackers that use light-weight CNNs like AlexNet^36^ are unable to extract both robust features and global context^21–23,25,37^. Many state-of-the-art trackers adpoted deeper neural networks like ResNet^38^ to address the performance issue^26–31,34,39,40^. In addition to improving the backbone networks, significant research has been conducted to enhance Siamese network-based frameworks. This includes the combination of various techniques such as DCF^41,42^, region proposal network (RPN) module^26,37,43,44^, template update module^44,45^, attention mechanism^24,34,35,46^, anchor-free mechanism^29–31,33^, and transformer mechanism^47–51^.

**Figure 1:**
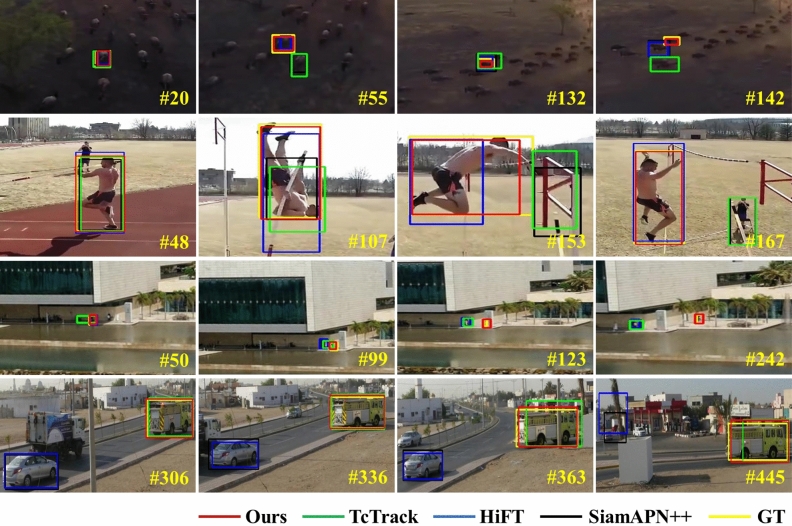
Qualitative comparison between state-of-the-arts. This figure shows the results of the proposed tracker AMST$\phantom{0}^2$ and three state-of-the-art trackers on some challenging video sequence (Animal2, Vaulting from DTB70, and Bike2, Truck1 from UAV123). The AMST$\phantom{0}^2$ tracker demonstrates superior performance over other algorithms by combining multi-level spatial and temporal context while adding the template update mechanism of feature-level.

Although general-purpose trackers have made significant advances, tracking in aerial environment such as UAVs demand faster processing while maintaining a certain level of performance. To meet these requirements, trackers that combine light-weight CNNs with various deep learning techniques have been proposed. In this context, SiamAPN utilized an anchor proposal network to refine anchors^52^. SiamAPN++ adopted an attentional aggregation network (AAN) to achieve robust aerial tracking in complex situations through the attention mechanisms of self- and cross-AANs^53^. Both SiamAPN and SiamAPN++ generated a small number of high-quality anchors to increase efficiency and performance of the tracker. HiFT^54^ and TCTrack^55^ are examples of recent aerial trackers that utilize light-weight CNNs and transformer architecture. HiFT addresses scale invariance by employing a hierarchical feature transformer that leverages global context from multiple CNN feature layers. On the other hand, TCTrack utilizes a transformer-based framework that incorporates temporal prior knowledge of search feature and similarity map, with modified light-weight CNNs that consider temporal information. Separating the use of multi-level spatial and temporal information can lead to a significant problem where high performance is only achieved in specific robust scenarios. For example, using multi-level spatial information may be robust to low-resolution and scale variation, while relying solely on temporal information may show better performance in dealing with deformation.

Integrating both spatial and temporal information can improve robustness and efficiency in complex scenarios. To achieve this, we propose an aggregated multi-level spatial and temporal context-based transformer (AMST$\phantom{0}^2$) architecture for robust aerial tracking. Our design includes an aggregation encoder based on a modified transformer encoder, and multi-level spatial and temporal encoders that capture useful contexts for an enhanced similarity map. The output of the multi-level spatial encoder is then simply injected into the output of the temporal encoder using the aggregation encoder. As a result, the output of the aggregation encoder is a robust embedding representation that can fully exploit the global contexts of multi-level spatial and the temporal contexts. The decoder focuses on the generation of more powerful refined similarity maps based on the output of the aggregation encoder. The multi-level spatial information included in the aggregation encoder deals with information highly relevant to small object tracking, which is a big issue in aerial tracking, and temporal information captures large changes in small objects. Additionally, the proposed model adopts a light-weight based backbone network. Using a light-weight backbone has an overall model size advantage over using a deep backbone when combined with various AI algorithms. As a result, these trackers can successfully solve the problem of tracking small objects in data obtained using UAVs while running in real time. Furthermore, in the existing method^55^, the updating of temporal information only at the feature level of the search can lead to high failure of the tracker due to inconsistency between the search and the template feature over time. Therefore, we further improve tracking performance by employing a template update network, which is the discrete temporal context update at the template level. As shown in Fig. 1, the proposed AMST$\phantom{0}^2$ achieves accurate and robust performance in complex scenarios.

The main contributions of this work can be summarized as follows:

- We propose a new aerial view tracking mechanism, which introduces the aggregation encoder that combines the encoder embedding representation of hierarchical feature of multi-level spatial contexts and temporal contexts feature within transformer structure.
- The proposed tracker not only applies temporal information at the search feature level and similarity map level, but also adopts the template update process at the template feature level as the discrete temporal context update for more robust tracking.
- We perform comprehensive experiments on various UAVs datasets for performance evaluation. The proposed tracker shows the achievement of state-of-the-art results compared to other aerial trackers with real-time processing.

## Related work

### Transformer in visual tracking

The transformer was first proposed by Vaswani et al. as a model for performing sequence-to-sequence tasks, such as machine translation^56^. This approach is based on the attention mechanism, which can efficiently capture the global information of the input sequence when generating the output sequence by focusing more on the most important part of the entire input sequence.

Recently, the transformer has been applied to vision tasks, including image classification^57^, object detection^58^, and action recognition^59^, in addition to natural language processing (NLP) fields. This approach has become increasingly popular due to its ability to incorporate both spatial and temporal context information in a flexible and efficient manner, enabling better tracking performance in various scenarios.

Most transformer-based trackers adopt a process of feeding the transformer with features extracted from the backbone network^47–50,54,55^. Inspired by the main idea of the transformer, TransT proposed a feature fusion network composed of an ego-context augmentation module with self-attention and a cross-feature augment module with cross-attention^47^. As a useful feature of the output of the feature fusion network, the final tracking result is obtained through classification and box regression processes. TrDiMP utilizes the DiMP model predictor and generates model weights by using the output features of the transformer encoder as training samples^48^. After that, the target model calculates the target score map by applying the predicted weights to the output features generated by the transformer decoder. TrDiMP incorporates a probabilistic IoUNet for bonding box regression and also introduces TrSiam, which formulates the proposed model into a Siamese-like pipeline. STARK, as proposed in^49^, is a tracker using an end-to-end transformer architecture based on DETR^58^. The model learns robust spatio-temporal representations by leveraging the global relationships in both spatial and temporal information through the encoder, which extracts discriminative spatio-temporal features that are fed into the decoder. Furthermore, this tracker eliminates the need for post-processing techniques such as cosine window or bounding box smoothing, thereby simplifying the existing tracking pipeline. ToMP predicts the weight of the convolutional kernel for object localization using a transformer-based model prediction module to overcome the limitations of the existing optimization-based target localization^50^. The transformer-based target model predictor can avoid unnecessary repetitive optimization and dynamically generate discriminative features using target information. AiATrack introduced an attention in attention (AiA) module that enhances appropriate correlations and suppresses ambiguous correlations in order to suppress the noise of the existing attention mechanism. By introducing a model update method that directly reuses previously encoded cached features, they propose a simplified tracking process that effectively utilizes short-term and long-term references, showing remarkable performance.

In addition, active and vibrant research has been conducted on transformer-based tracking methods that adopt a lightweight backbone for aerial tracking^54,55^. Unlike the trackers mentioned above, the research on trackers in which the backbone is replaced with transformers instead of existing CNNs also shows remarkable performance^60,61^.

### Multi-level spatial and temporal information-based visual tracking

Incorporating both spatial and temporal information is crucial for enhancing performance in the field of object tracking. There are many trackers that use multi-level spatial feature to extract the relationship between the template and the current search region according to the spatial dimension^12,26,29,30,54^. The tracker using multi-scale features has the advantage of being able to robustly track the localization of objects of various scales. Dynamic template-based trackers, such as Updatenet^45^ and SiamTOL^44^, have been developed to enhance tracking performance by utilizing temporal information. In particular, TCTrack introduced a tracking method considering the temporal contexts of two levels, including the search feature level and the similarity map level^55^. Trackers that take into account temporal information can achieve robust performance by capturing changes in the state of the object across frames. However, when using multi-level spatial and temporal information separately, there is a problem that the complementary advantages of the two information cannot be utilized. To address this limitation, a method has been introduced to improve the robustness of the tracker by integrating spatial and temporal information through simultaneous learning with the transformer, as demonstrated in the STARK tracker^49^.

### Aerial visual tracking

Due to the technological advancements in UAVs equipped with visual tracking capabilities, aerial tracking has been widely applied in sectors such as aviation, agriculture, transportation, and defense^1–3^. One significant challenge in aerial tracking arises from image distortion caused by UAV flight vibrations and complex environments. Specially, in aerial tracking, when UAVs flying at a high altitude captures an object on the ground, it is difficult to extract rich features due to the small size of the object. While deep learning-based trackers have demonstrated superiority on various UAV datasets, the limited resources of aerial platforms hinder the use of heavy models and limit tracking performance improvement. To address these challenges, several specialized trackers have been developed using different UAV datasets.

AutoTrack is a DCF-based tracker that automatically tunes the hyperparameters of the space-time regularization, demonstrating high performance on CPU^62^. COMET improves tracking accuracy by proposing context-aware IoU-guided tracker that utilizes a multi-task two-stream network for small object tracking and an offline reference proposal generation strategy^63^. Additionally, adopting an anchor proposal network to generate high-quality anchors for light-weight Siamese network-based trackers has shown excellent aerial tracking performance^52,53^. Moreover, employing a transformer to the light-weight Siamese network backbone has resulted in notable progress by enhancing the correlation map^54,55^.

The development of miniaturized embedded AI computing platforms offers a promising alternative to dedicated server GPUs, enabling continuous research and practical use in future aerial tracking endeavors.

## Proposed method

**Figure 2:**
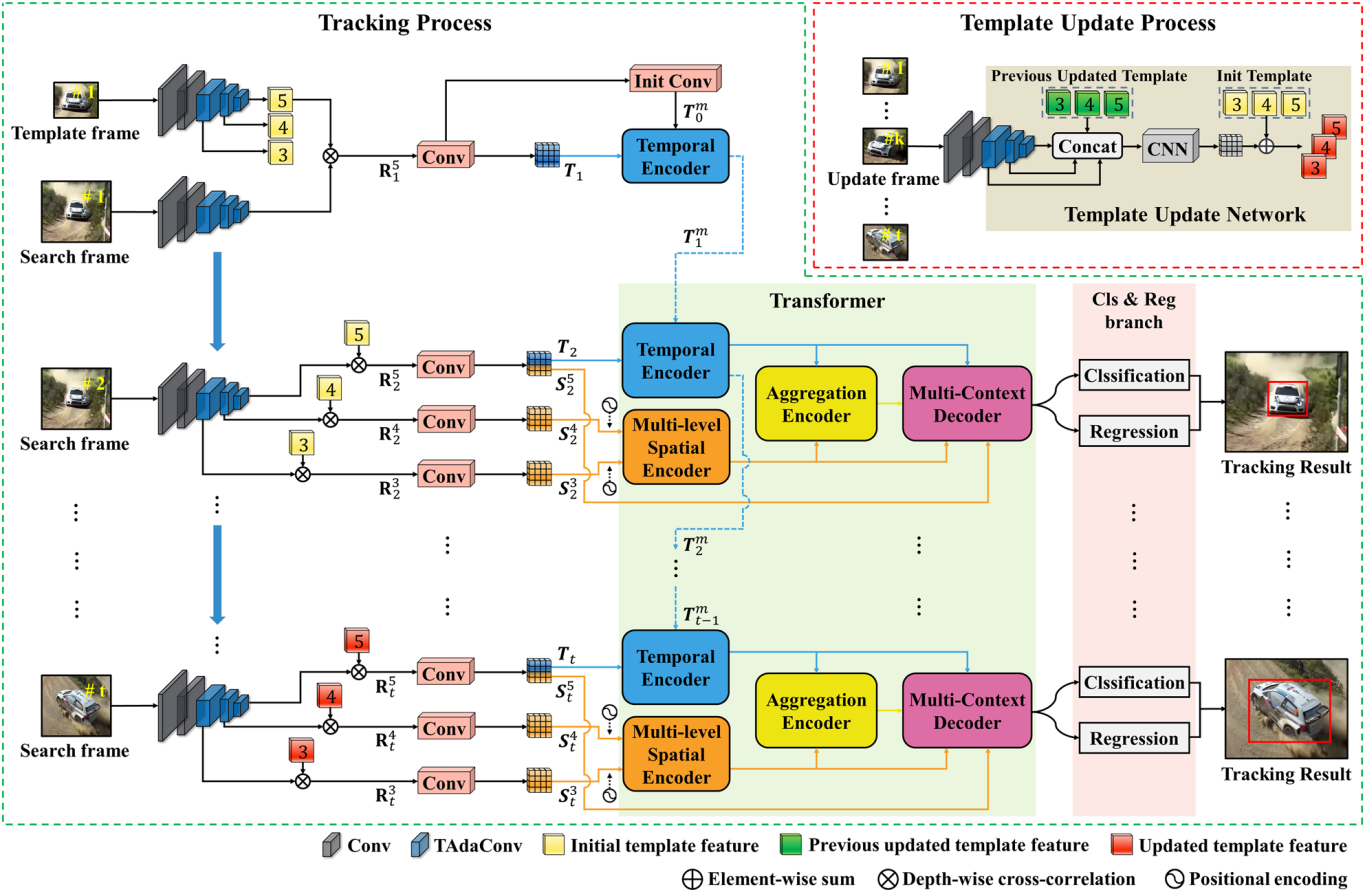
The overall tracking process of the proposed tracker. The AMST$\phantom{0}^2$ tracker is composed of four main components: a Siamese feature extractor, template update network, transformer, and classification and regression network. The transformer module consists of multi-level spatial, temporal, and aggregation encoders, along with a multi-context decoder. The multi-level spatial encoder takes the similarity map generated from the 3rd and 4th layer features as input, while the temporal encoder uses the similarity map generated from the 5th layer features and the output of the previous temporal encoder (indicated by the blue dotted line) as input. The aggregation encoder receives the outputs of multi-level spatial and temporal encoders as inputs. The multi-context decoder uses the outputs of all encoders and the similarity map generated with 5th layer features as inputs. Furthermore, the template update process incorporates an update patch, previous template features, and initial template features. This process is executed either during each specific frame or under certain conditions to update the template.

In this section, we present the AMST$\phantom{0}^2$ tracker for aerial tracking, which utilizes an aggregated multi-level spatial and temporal context-based transformer. The proposed tracker consists of four sub modules: (1) the Siamese feature extraction network, (2) template update network, (3) transformer module (which includes the multi-level spatial encoder, temporal encoder, aggregation encoder, and multi-context decoder), and (4) classification and regression network. To provide a clear comparison with existing tracking algorithms, we introduce baseline algorithms that utilize the multi-level spatial encoder, temporal encoder, and template update network. We then propose an extension to these baseline algorithms by adopting an aggregation encoder that combines the representations learned by the multi-level spatial and temporal encoders, along with a modified decoder for tracking. A visual representation of our method can be seen in Fig. 2, and we provide further details on the approach below.

### Feature extraction network

As a feature extraction backbone, deep CNNs such as GoogLeNet^64^, MobileNet^65^, and ResNet^38^ have been widely used in various trackers. However, the heavy computation requirements limit their employment in embedded platforms such as UAVs.

To solve this problem, we transformed a light-weight feature extractor such as AlexNet with additional convolution layers into online temporally adaptive convolution (TAdaConv)^66^, inspired by^55^. TAdaConv considers the temporal context at the search feature level. A typical convolutional layer shares learnable weights and bias in the entire tracking sequence. On the other hand, the parameters of the online convolution layer are calculated by a calibration factors that are varied for each frame and learnable weights and bias. As a result, it is possible to extract features that contain temporal information at the feature level using the convolutional weight dynamically calibrated by the previous frame. Since TAdaConv is calibrated using global descriptors of the feature in the previous frames, the tracking performance with temporal adaptive convolutional network (TAdaCNN) improves remarkably despite a diminutive frame rate drop. For more details on how to transform a standard convolution layer into TAdaConv, please refer to^55,66^.

Utilizing both low-level and high-level convolution layers’ features improves tracking accuracy. Therefore, using TAdaCNN $\phi$ as the backbone, multi-level spatial information is obtained by calculating the similarity map using the hierarchical features of the TAdaCNN’s multi-layer at the *t*-th frame.

1 $$\textbf{R}_t^i=\phi _t^i\left(\textrm{Z}_t\right) \circledast \phi _t^i\left(\textrm{X}_t\right) ,\quad i=3,4,5,$$

where $\textrm{Z}$ and $\textrm{X}$ represent template and search image respectively. $\circledast$ denotes depth-wise cross correlation and $\phi _{\textrm{t}}^{i}\left( \cdot \right)$ represents the *i*-th convolution layer of TAdaCNN in the *t*-th frame. To exploit multi-layer deep features, we extract features after transforming the last three convolution layers of the backbone to TAdaConv. Finally, the similarity map ${\textbf{R}}_{t}^{3}\in {\mathbb {R}}^{H\times W\times C}$, ${\textbf{R}}_{t}^{4}\in {\mathbb {R}}^{H\times W\times C}$, and ${\textbf{R}}_{t}^{5}\in {\mathbb {R}}^{H\times W\times C}$ can be obtained by using multi-layer deep features.

### Transformer encoder

**Figure 3:**
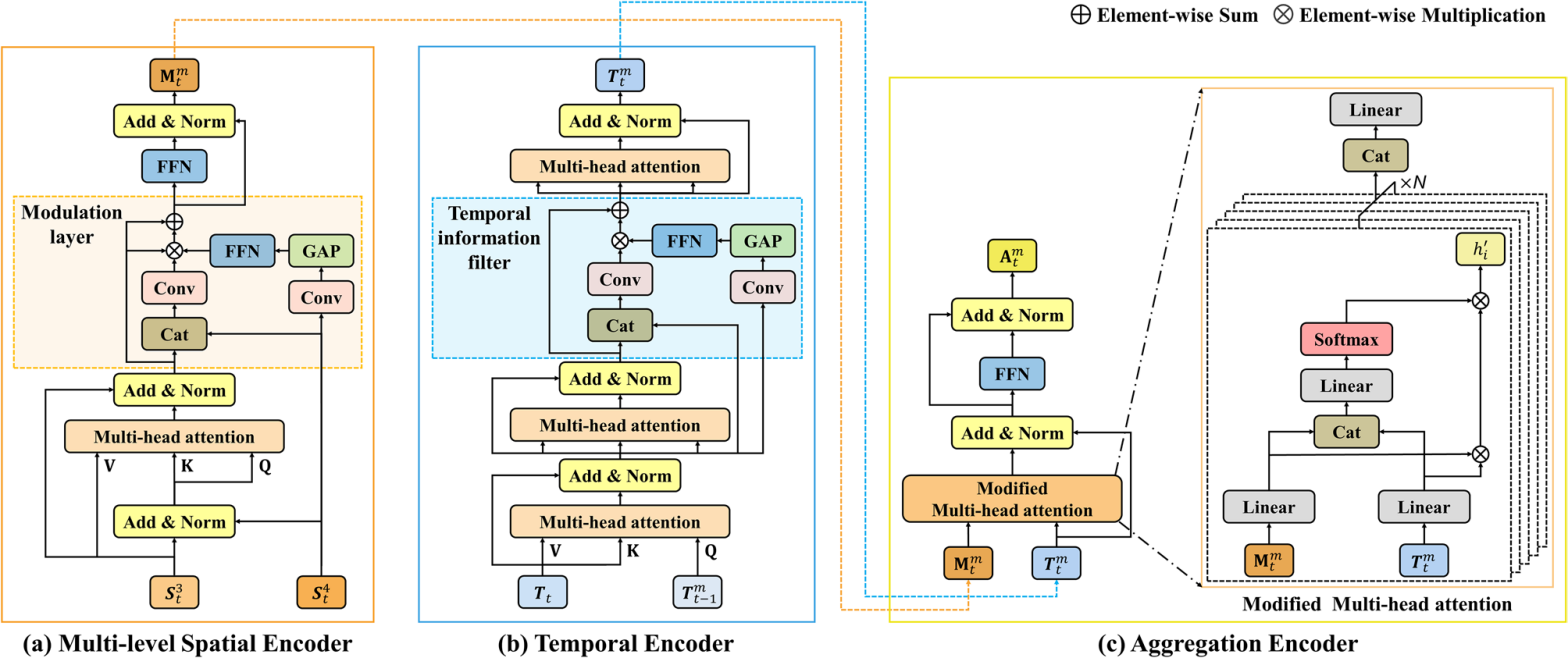
Architecture of the proposed transformer encoder. The proposed encoder consists of three components: a multi-level spatial encoder, a temporal encoder, and an aggregation encoder.

The similarity maps calculated using the hierarchical features of multi-level layer of backbone are pre-processed before being fed into multi-level spatial and temporal encoders. The architecture of the proposed transformer encoder is shown in Fig. 3. First, the similarity maps ${\textbf{R}}_{t}^{3}$, ${\textbf{R}}_{t}^{4}$ and ${\textbf{R}}_{t}^{5}$ obtained from *t*-th frame are passed through the convolutional layer. Afterwards, the refined similarity maps ${{\varvec{T}}}_{t}\in {\mathbb {R}}^{HW\times C}$, ${{\varvec{S}}}_{t}^{3}\in {\mathbb {R}}^{HW\times C}$, ${{\varvec{S}}}_{t}^{4}\in {\mathbb {R}}^{HW\times C}$, and ${{\varvec{S}}}_{t}^{5}\in {\mathbb {R}}^{HW\times C}$ can be obtained using reshape operation ( ${{\varvec{T}}}_{t}$ can be obtained by copying ${{\varvec{S}}}_{t}^{5}$, such that ${{\varvec{T}}}_{t}$ = ${{\varvec{S}}}_{t}^{5}$).

The attention mechanism is a crucial component in a standard transformer. It involves using the query, key, and value represented as ${\textbf{Q}},{\textbf{K}},$ and ${\textbf{V}}$, respectively. The attention function in a standard transformer is typically defined as scale dot-product attention, which can be expressed as:

2 $$\begin{aligned} \textrm{Attention}\left( {\textbf{Q}},{\textbf{K}},{\textbf{V}}\right) =\textrm{softmax}\left( \frac{{\textbf{Q}}{\textbf{K}}^{\textrm{T}}}{\sqrt{d_{k}}}\right) {\textbf{V}}, \end{aligned}$$

where $1/\sqrt{d_{k}}$ is a scaling factor to control the softmax distribution and avoid gradient vanishing problem. By extending the attention module to multiple heads, the model can extract representations in multiple subspaces as follows:

3 $$\begin{aligned} \text {MultiHead}\left( {\textbf{Q}}, {\textbf{K}}, {\textbf{V}}\right){} & {} =\text {Concat}\left( {\textbf{h}}_{1},\ldots ,{\textbf{h}}_{N}\right) {\textbf{W}}^{{\textbf{O}}},\nonumber \\ {\textbf{h}}_{j}{} & {} =\text {Attention}\left( \textbf{QW}_{j}^{{\textbf{Q}}},\,\textbf{KW}_{j}^{{\textbf{K}}},\,\textbf{VW}_{j}^{{\textbf{V}}}\right) , \end{aligned}$$

where ${\textbf{W}}_{j}^{{\textbf{Q}}}\in {\mathbb {R}}^{C\times C/N}$, ${\textbf{W}}_{j}^{{\textbf{K}}}\in {\mathbb {R}}^{C\times C/N}$, ${\textbf{W}}_{j}^{{\textbf{V}}}\in {\mathbb {R}}^{C\times C/N}$, and ${\textbf{W}}^{{\textbf{O}}}\in {\mathbb {R}}^{C\times C}$ are learnable weight matrices, $\textrm{Concat}(\cdot)$ represents the concatenation and $N$ is the number of attention head.

#### Multi-level spatial encoder

Cao et al. utilized a combination of multi-level spatial information to fully explore inter-dependencies between hierarchical features^54^. Specifically, with learnable position encoding, ${{\varvec{S}}}_{t}^{3}$ and ${{\varvec{S}}}_{t}^{4}$ are combined using addition and a normalization to obtain ${\textbf{M}}_{t}^{1}$, i.e., ${\textbf{M}}_{t}^{1}=\textrm{Norm}\left( {{\varvec{S}}}_{t}^{3}+{{\varvec{S}}}_{t}^{4}\right)$, which is then fed into a multi-head attention layer to obtain ${\textbf{M}}_{t}^{2}$ using the equation in (3).

4 $$\begin{aligned} {\textbf{M}}_{t}^{2}=\textrm{MultiHead}\left( {\textbf{M}}_{t}^{1},\,{\textbf{M}}_{t}^{1},\,{{\varvec{S}}}_{t}^{3}\right) . \end{aligned}$$

As shown in (4), by considering the global context of ${{\varvec{S}}}_{t}^{3}$ and ${{\varvec{S}}}_{t}^{4}$ and learning the inter-dependencies of the two feature maps, ${\textbf{M}}_{t}^{2}$ is enhanced to a high-resolution feature map. Thereafter, ${\textbf{M}}_{t}^{3}$ can be obtained by add operation and normalization layer, i.e., ${\textbf{M}}_{t}^{3}=\textrm{Norm}\left( \mathbf {{M}}_{t}^{2}+{{\varvec{S}}}_{t}^{3}\right)$. To fully explore the inter-dependencies between ${\textbf{M}}_{t}^{3}$ and ${{\varvec{S}}}_{t}^{4}$, we adopt a modulation layer. The modulation layer can efficiently exploit the internal spatial information of between ${\textbf{M}}_{t}^{3}$ and ${{\varvec{S}}}_{t}^{4}$, the output ${\textbf{M}}_{t}^{4}$ of modulation layer can be expressed as:

5 $$\begin{aligned} {\textbf{M}}_{t}^{4}{} & {} ={\textbf{M}}_{t}^{3}\,+\,\gamma *{\textbf{w}}*{\textbf{M}}_{t}^{3}, \nonumber \\ {\textbf{w}}{} & {} ={\mathcal {F}}\left( \textrm{Concat}\left( {\textbf{M}}_{t}^{3},\, S _{t}^{4}\right) \right) \,*\,\textrm{FFN}\left( \textrm{GAP}\left( {\mathcal {F}}\left( {{\varvec{S}}}_{t}^{4}\right) \right) \right) , \end{aligned}$$

where $$\textrm{FFN}\left( \cdot \right)$$ denotes a feed-forward network (FFN), $$\textrm{GAP}\left( \cdot \right)$$ denotes a global average pooling (GAP), and $$\gamma$$ and $${\mathcal {F}}\left( \cdot \right)$$ represent learning weight and convolution layer, respectively. The final output $${\textbf{M}}_{t}^{m}\in {\mathbb {R}}^{HW\times 
C}$$ of multi-level spatial encoder can be expressed as:

6 $$\begin{aligned} {\textbf{M}}_{t}^{m}=\textrm{Norm}\left( {\textbf{M}}_{t}^{4}+\textrm{FFN}\left( {\textbf{M}}_{t}^{4}\right) \right) . \end{aligned}$$

The compressed embedding features of the multi-level spatial encoder not only effectively discriminate objects from the scale variation scenario, but are also robust to small object detection. The multi-level spatial encoder is shown in Fig. 3a.

#### Temporal encoder

Aside from using temporal information at the feature level, Cao et al. refined the similarity map using temporal prior knowledge by integrating both the previous knowledge and the current information at the similarity level^55^. The temporal context-based encoder structure is composed of three multi-head attention layers and one temporal information filter. The temporal encoder is shown in Fig. 3b. Given the previous prior knowledge ${{\varvec{T}}}_{t-1}^{m}$ and the current similarity map ${{\varvec{T}}}_{t}$ as inputs of the encoder, ${{\varvec{T}}}_{t}^{1}$ can be obtained using the first multi-head attention layer.

7 $$\begin{aligned} {{\varvec{T}}}_{t}^{1}=\textrm{MultiHead}\left( {{\varvec{T}}}_{t-1}^{m},\,{{\varvec{T}}}_{t},\,{{\varvec{T}}}_{t}\right) . \end{aligned}$$

Then, ${{\varvec{T}}}_{t}^{2}$ is obtained by normalizing after adding ${{\varvec{T}}}_{t}$ and ${{\varvec{T}}}_{t}^{1}$, i.e., ${{\varvec{T}}}_{t}^{2}=\textrm{Norm}\left( {{\varvec{T}}}_{t}+{{\varvec{T}}}_{t}^{1}\right)$. In the same way as in (7), ${{\varvec{T}}}_{t}^{3}$ is obtained using ${{\varvec{T}}}_{t}^{2}$ as the input of the second multi-head attention layer.

8 $$\begin{aligned} {{\varvec{T}}}_{t}^{3}=\textrm{MultiHead}\left( {{\varvec{T}}}_{t}^{2},\,{{\varvec{T}}}_{t}^{2},\,{{\varvec{T}}}_{t}^{2}\right) . \end{aligned}$$

After that, ${{\varvec{T}}}_{t}^{4}$ can be obtained by add operation and normalization layer, i.e., ${{\varvec{T}}}_{t}^{4}=\textrm{Norm}\left( {{\varvec{T}}}_{t}^{2}+{{\varvec{T}}}_{t}^{3}\right)$. During tracking, the degraded temporal context occurs due to various noises. Hence, the unnecessary context may be included, which degrades tracker performance when temporal information of the entire frame is exploited. To solve this problem, the temporal information filter can be obtained by feeding the global descriptor of ${{\varvec{T}}}_{t}^{2}$, which is the result of GAP into the FFN. The temporal information filter and the filtered information ${{\varvec{T}}}_{t}^{f}$ can be expressed as:

9 $$\begin{aligned} {{\varvec{T}}}_{t}^{f}{} & {} ={{\varvec{T}}}_{t}^{4}+\gamma *f *{\mathcal {F}}\left( \textrm{Concat}\left( {{\varvec{T}}}_{t}^{2},\,{{\varvec{T}}}_{t}^{4}\right) \right) , \nonumber \\ f{} & {} =\textrm{FFN}\left( \textrm{GAP}\left( {\mathcal {F}}\left( {{{\varvec{T}}}_{t}^{2}}\right) \right) \right) , \end{aligned}$$

where *f* is the temporal information filter. The temporal knowledge of the *t*-th frame ${{\varvec{T}}}_{t}^{m}\in {\mathbb {R}}^{HW\times C}$ as the final output of the temporal encoder can be expressed as:

10 $$\begin{aligned} {{\varvec{T}}}_{t}^{m}=\textrm{Norm}\left( {{\varvec{T}}}_{t}^{f}+\textrm{MultiHead}\left( {{\varvec{T}}}_{t}^{f},\,{{\varvec{T}}}_{t}^{f},\,{{\varvec{T}}}_{t}^{f}\right) \right) , \end{aligned}$$

where $\textrm{Norm}\left( \cdot \right)$ denotes normalization layer. Notably, the first frame has a problem in that there is no distinguishing characteristic of the previous frame. Therefore, by convolution operation, the initial similarity map is set to ${{\varvec{T}}}_{0}^{m}={\mathcal {F}}_{init}\left( {{\varvec{T}}}_{1}\right)$, where ${\mathcal {F}}_{init}\left( \cdot \right)$ represents the initial convolution layer.

#### Aggregation encoder

In order to improve tracking performance by utilizing integrated multi-level spatial information and temporal information, we propose an aggregation encoder that aggregates the outputs of the multi-level spatial and temporal encoders. The aggregation encoder modifies the multi-head attention layer of the standard encoder, allowing the output of the multi-level spatial encoder to be injected into the output of the temporal encoder. The attention weight for the aggregation encoder can be expressed as follows, given the outputs ${\textbf{M}}_{t}^{m}$ and ${{\varvec{T}}}_{t}^{m}$ of each encoder:

11 $$\begin{aligned} \alpha _{j}{} & {\phantom{0}} = \textrm{Concat}\left( {\textbf{M}}_{t}^{m}{\textbf{W}}_{j}^{{\textbf{M}}},\,{{\varvec{T}}}_{t}^{m}{\textbf{W}}_{j}^{{{\varvec{T}}}}\right) ,\nonumber \\ w_{j}{\phantom{0}} & {\phantom{0}} =\textrm{softmax}\left( \alpha _{j}{\textbf{W}}_{j}^{\alpha }\right) , \end{aligned}$$

where ${\textbf{W}}_{j}^{{\textbf{M}}}\in {\mathbb {R}}^{C\times C/N}$, ${\textbf{W}}_{j}^{{{\varvec{T}}}}\in {\mathbb {R}}^{C\times C/N}$, ${\textbf{W}}_{j}^{\alpha }\in {\mathbb {R}}^{2C\times C/N}$ are learnable weight of the linear layer and *j* is the index of the head. According to (11), the output of the $j\text{-th}$ head and the output *H* of modified multi-head attention layer can be expressed as by:

12 $$\begin{aligned} h_{j}^{'}{} & {} =w_{j}*\left( {\textbf{M}}_{t}^{m}{\textbf{W}}_{j}^{{{\varvec{M}}}}*{{\varvec{T}}}_{t}^{m}{\textbf{W}}_{j}^{{{\varvec{T}}}}\right) ,\nonumber \\ H{} & {} =\textrm{Concat}\left( {\textbf{h}}^{'}_{1},\ldots ,{\textbf{h}}^{'}_{N}\right) {\textbf{W}}^{O}, \end{aligned}$$

where ${\textbf{W}}^{O}\in {\mathbb {R}}^{C\times C}$ are learnable weight matrices and $N$ is the number of attention head. Afterwards, ${\textbf{A}}_{t}^{1}$ can be obtained by using add operation and normalization layer, i.e., ${\textbf{A}}_{t}^{1}=\textrm{Norm}\left( {{\varvec{T}}}_{t}^{m}+H\right)$. Finally, the output ${\textbf{A}}_{t}^{m}$ of the aggregation encoder can be obtained by:

13 $$\begin{aligned} {\textbf{A}}_{t}^{m}=\textrm{Norm}\left( {\textbf{A}}_{t}^{1}+\textrm{FFN}\left( {\textbf{A}}_{t}^{1}\right) \right) . \end{aligned}$$

The output of the aggregation encoder integrates multi-level spatial and temporal information to generate more powerful features omplex scenarios. The detailed structure of aggregation encoder is shown in Fig. 3c.

### Transformer decoder

**Figure 4:**
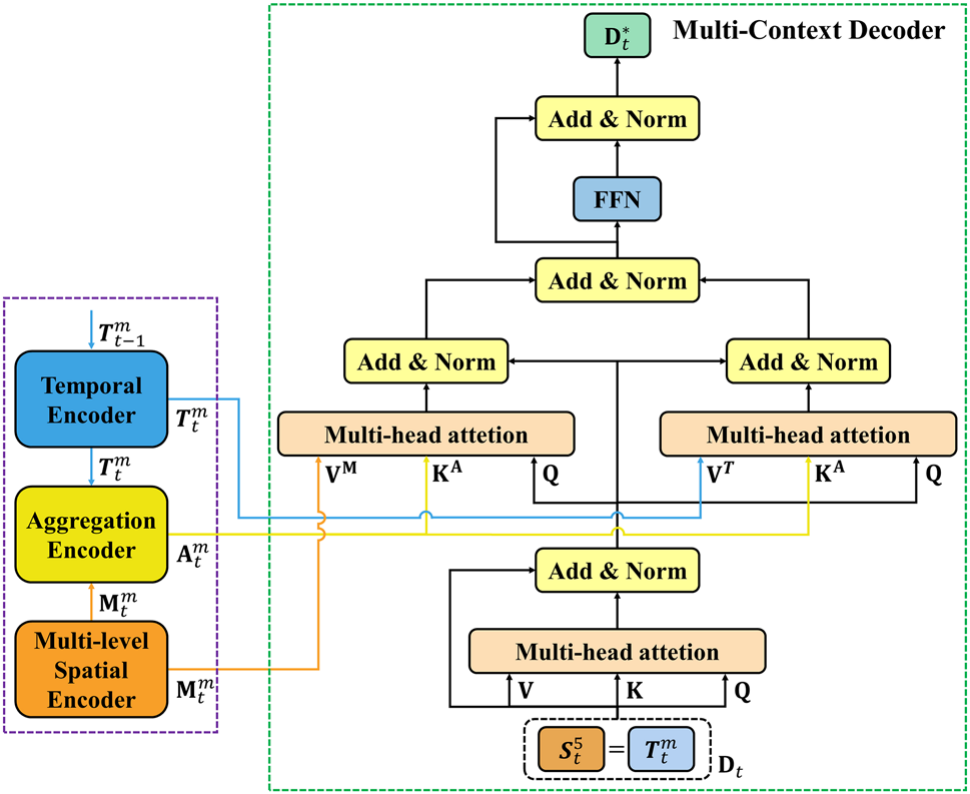
Architecture of the proposed transformer decoder. The proposed decoder aims to refine the similarity map using multiple context-based information and is composed of three multi-head attention modules.

We propose a multi-context decoder to utilize both high-resolution and low-resolution information, and further exploit the interrelation between current spatial features and temporal knowledge. The proposed multi-context decoder introduces a structure that integrates the refined multi-context features using the outputs of the multi-level spatial and temporal encoders. Therefore, we adopt three multi-head attention differently from the decoder structure of the standard transformer. Also, after the first multi-head attention, the output of the aggregation encoder was used for the key, and the output of the multi-level spatial and temporal encoders were used for the value, respectively. Therefore, the proposed method not only maintains the feature information of each of the multi-level spatial and the temporal encoders, but also obtains the feature with increased attention at a corresponding location containing the multi-context information based on the valid information of the location containing the aggregated multi-context information of the aggregation encoder. The positional encoding of the multi-level spatial encoder is used to distinguish each location on the feature map. However, in order to avoid direct influence on the multi-context-based transformed features, the decoder is designed without positional encoding and implicitly receives the positional information of the multi-level spatial encoder^54^. The multi-context decoder is shown in Fig. 4.

The current low-resolution similarity map ${{\varvec{S}}}_{t}^{5}$ and ${{\varvec{T}}}_{t}$ are the same similarity map and are denoted as ${\textbf{D}}_{t}$, the normalized result after adding to ${\textbf{D}}_{t}$ passed through multi-head attention is as follows:

14 $$\begin{aligned} {\textbf{D}}_{t}^{1}=\textrm{Norm}\left( {\textbf{D}}_{t}+\textrm{MultiHead}\left( {\textbf{D}}_{t},\,{\textbf{D}}_{t},\,{\textbf{D}}_{t}\right) \right) . \end{aligned}$$

The outputs of calculating the two multi-head attentions using both ${\textbf{D}}_{t}^{1}$ and the outputs of the encoders is then normalized after adding to ${\textbf{D}}_{t}^{1}$ is expressed as:

15 $$\begin{aligned} {\textbf{D}}_{t}^{2}{} & {} =\textrm{Norm}\left( {\textbf{D}}_{t}^{1}+\textrm{MultiHead}\left( {\textbf{D}}_{t}^{1},\,{\textbf{A}}_{t}^{m},\,{\textbf{M}}_{t}^{m}\right) \right) , \nonumber \\ {\textbf{D}}_{t}^{3}{} & {} =\textrm{Norm}\left( {\textbf{D}}_{t}^{1}+\textrm{MultiHead}\left( {\textbf{D}}_{t}^{1},\,{\textbf{A}}_{t}^{m},\,{{\varvec{T}}}_{t}^{m}\right) \right) , \end{aligned}$$

where ${\textbf{D}}_{t}^{2}$ is the result of set the key and value to ${\textbf{A}}_{t}^{m}$ and ${\textbf{M}}_{t}^{m}$, respectively, and ${\textbf{D}}_{t}^{3}$ is the result of set the key and value to ${\textbf{A}}_{t}^{m}$ and ${{\varvec{T}}}_{t}^{m}$, respectively. The final result ${\textbf{D}}_{t}^{*}$ of the transformer containing multi-context information can be obtained by using ${\textbf{D}}_{t}^{2}$ and ${\textbf{D}}_{t}^{3}$ obtained from (15).

16 $$\begin{aligned} {\textbf{D}}_{t}^{4}{} & {} =\textrm{Norm}\left( {\textbf{D}}_{t}^{2}+{\textbf{D}}_{t}^{3}\right) , \nonumber \\ {\textbf{D}}_{t}^{*}{} & {} =\textrm{Norm}\left( {\textbf{D}}_{t}^{4}+\textrm{FFN}\left( {\textbf{D}}_{t}^{4}\right) \right) . \end{aligned}$$

### Template update

Despite using temporal context information through TAdaCNN, the updating of temporal information only at the feature level of the search can lead to high failure of the tracker due to inconsistency between the search and the template feature over time. In addition, when updating a template using backbone network, the information of the initial template which is a non-contaminated sample can be lost and violates the criteria of visual tracking to track arbitrary object using an initial template. We adopt the template update network as a feature fusion network^44^ to combine the features of the initial template and the update sample and can be seen in Fig. 2.

Given the template and the update sample in the *k*-th frame, the updated template $\hat{\textrm{Z}}_{k}$ using the template update network is calculated as:

17 $$\begin{aligned} \hat{\textrm{Z}}_{k}^{i}=\psi _{k}^{i}\left( \textrm{Concat}\left( \tilde{\textrm{Z}}_{k}^{i},\,\, \phi _{k}^{i}\left( \textrm{U}_{k}\right) \right) \right) +\phi _{1}^{i}\left( \textrm{Z}_{1}\right) ,\quad i=3,4,5, \end{aligned}$$

where $\textrm{Z}_{1}$ and $\textrm{U}_{k}$ denotes the initial template and the *k*-th frame updated image, respectively. $\tilde{\textrm{Z}}_{k}^{i}$ and $\phi _{1}^{i}\left( \textrm{Z}_{1}\right)$ respectively represent the previous updated template and the initial template feature of the first frame. $\psi _{k}^{i}\left( \cdot \right)$ represents the template update network. $\tilde{\textrm{Z}}_{k}^{i}$ is initialized to $\phi _{1}^{i}\left( \textrm{Z}_{1}\right)$ in the first updating process. The template update network consists of three 1$\times$1 convolutional layers with different channels of *C*, *C*/2, and *C*. Each of the first two convolutional layers is followed by a ReLU. We update the template every $\delta$ frames or when the confidence score is lower than the threshold $\tau$. The template update network can learn powerful representations of object appearance changes and can prevent tracking failure due to extreme drift over time.

### Network training loss

The proposed loss function consists of two branches for classification and regression tasks, similar to the HiFT tracker^54^. The first classification branch computes the foreground and background scores of a given location, while the second branch measures the distance contrast between the location and the center of the ground-truth to remove low-quality boxes. For regression, a linear combination of the L1-norm and the complete-IoU (CIoU)^67^ is used. The regression loss can be formulated as:

18 $$\begin{aligned} L_{loc}&=\sum_{j}\left[\lambda_{I}\cdot\left(1-IoU\left(b_{j},b^{gt}\right)\right)+\lambda_{C}\cdot\left(\frac{\rho^{2}\left(c_{j},c^{gt}\right)}{d^{2}}+\alpha\upsilon\right)+\lambda_{\mathrm{L}1}\cdot \mathrm{L}1\left(b_{j},b^{gt}\right)\right],\\ \upsilon&=\frac{4}{\pi^{2}}\left(arctan\frac{w^{gt}}{h^{gt}}-arctan \frac{w_{j}}{h_{j}}\right)^{2},\,\,\,\,\alpha=\frac{\upsilon}{1-IoU\left(b_{j},b^{gt}\right)+\upsilon}, \end{aligned}$$

where ${\textbf{b}}_{j}$ is the *j*-th predicted bounding box and ${\textbf{b}}^{gt}$ is its corresponding ground-truth box,  *c*_*j*_ and *c*^*gt*^ respectively represent the center of the predicted and ground-truth boxes, $\rho \left( \cdot \right)$ represents Euclidean distance, and *d* is the diagonal length of the box covering the predicted bounding box and the ground-truth box, and $\upsilon$ represents the correspondence between the aspect ratios of the predicted bounding box and the ground-truth box, and $\alpha$ is a positive trade-off parameter, which controls the balance between non-overlapping cases and overlapping cases, and $\lambda _{I}=1$, $\lambda _{C}=0.5$, and $\lambda _{L1}=0.5$ are the regularization parameters in our experiments.The total loss function can be expressed as:

19 $$\begin{aligned} L_{total}=\lambda _{1}\cdot L_{cls1}+\lambda _{2}\cdot L_{cls2}+\lambda _{3}\cdot loc, \end{aligned}$$

where $\lambda _{1}=1$, $\lambda _{2}=1$, and $\lambda _{3}=1.2$ are the regularization parameters in our experiments.

The feature extractor of the proposed model includes a Siamese network and a template update network to control features online. However, training the network with only a total loss can lead to over-fitting and a dilemma in balancing the function between the Siamese network and the template update network. To address this issue, we adopt a multi-aspect loss training method^44^. The multi-aspect training loss includes three aspects. Firstly, $L_{template}$ loss is based on the template sample and the search region to allow the network to track like an existing Siamese tracker using the template. Secondly, $L_{update}$ loss is obtained using the update sample and the search region, which can also be regarded as a template sample, resulting in a complementary sample data augmentation effect. Thirdly, $L_{overall}$ loss is obtained by using the updated template, which is the output of the template update network, and the search area to learn to track the location of an object using the updated template information. Finally, $L_{final}$ loss is expressed as:

20 $$\begin{aligned} L_{final}=L_{template}+L_{update}+L_{overall}, \end{aligned}$$

where $L_{template}$, $L_{update}$, and $L_{overall}$ are constructed as $L_{total}$ of (19) loss obtained using template sample, update sample, and updated template feature, respectively.

## Experimental results

In this section, we conducted comprehensive experiments of the proposed tracker AMST$\phantom{0}^2$ on various UAVs datasets including DTB70^68^, UAV123^69^, UAV123@10fps^69^, UAV20L^69^, UAVTrack112$\_$L^70^, VisDrone-SOT2020^71^ and UAVDT^72^. To evaluate the performance of the SOTA visual tracking method, we quantitatively compared the proposed tracker with 51 existing top trackers. The existing methods include light-weight trackers^5–12,16,21–23,26,32,37,52–55,62,73–76^ trackers and the deep trackers^26–31,33,39,40,46–51,77,78^. For fair comparison, we used Siamese network-based trackers for all the lightweight backbone such as AlexNet. In all experiments, we used publicly available codes or results provided by the original author.

### Implementation details

#### Training

In the training phase, AMST$\phantom{0}^2$ was trained on ImageNet VID^19^, COCO^79^, GOT-10K^80^, and LaSOT^81^ datasets. We exploited three samples for training. We used the same patch size 127 $\times$ 127 for both template and update, and used the search patch of size 287 $\times$ 287. Our backbone is an AlexNet with the last three layers converted by TAdaConv and initialized with pre-trained weights from ImageNet. For efficient learning of the temporal context of TAdaConv, we used one search patch in a half and two search patches in one third for the entire epoch, respectively, and three search patches for the remaining epochs. The transformer architecture consists of one multi-level spatial encoder layer, one temporal encoder layer, one aggregation encoder layer and two multi-context decoder layers. Our whole networks are trained with stochastic gradient descent (SGD) with momentum and weight decay of 0.9 and 0.0001, respectively. The batch size was 180 and the network was trained for 100 epochs. For the first 20 epochs, the layers of backbone are frozen and the remaining epochs fine-tune the last three layers. We used a warm-up learning rate from 0.005 to 0.01 in the first 10 epochs and a decreasing learning rate from 0.01 to 0.00005 in log space in the remaining epochs. The training process was conducted with two NVIDIA RTX 3090 GPUs.

#### Testing

In the inference phase, to obtain the initial temporal prior knowledge, we calculated the correlation between the template and search patches using only the initial frame. Afterwards, the smooth object tracking was possible by continuously matching the feature of the search area cropped based on the object position of the previous frame with the template feature obtained in the initial frame or the updated template feature through the template update network. The threshold $\tau$ of the template update process was set to 0.8. In addition, $\delta$ was set to 50 for short-term aerial tracking datasets such as DTB70 and 150 for long-term aerial data sets such as UAV123. In order to smooth the motion of the object, the cosine window and the scale change penalty are applied for the predicted box to eliminate the boundary outliers and minimize the large changes in size and ratio^5,37^. After that, by selecting the prediction box with the best score, the size of the bounding box is updated by linear interpolation. Fig. 2 shows a whole tracking process, where our tracker operates on a single NVIDIA RTX 3090 GPU for real-time tracking.

### Evaluation metrics

We employed One Pass Evaluation (OPE)^69,82^ to evaluate the proposed method. OPE is based on two metrics: (1) precision and (2) success rate.

The precision exploits the center location error (CLE) between the predicted bounding box and the ground-truth box.

21 $$\begin{aligned} \textbf{CLE}=\left\| c_{t}-c_{t}^{gt}\right\| , \end{aligned}$$

where $c_{t}$ and $c_{t}^{gt}$ respectively represent the center of the *t*-th predicted and ground-truth bounding boxes, and $\left\| \cdot \right\|$ is the Euclidean distances. The precision plot displays the percentage of frames where the center location error is below a specific threshold. A threshold of 20 pixels is utilized to evaluate and rank the trackers.

The success rate is calculates overlap as the IOU between the predicted and ground-truth bounding boxes. The overlap ratio $\textbf{OR}_{t}$ in the *t*-th frame is expressed as:

22 $$\begin{aligned} \textbf{OR}_{t}=\frac{\left| b_{t}\cap b_{t}^{gt}\right| }{\left| b_{t}\cup b_{t}^{gt} \right| }, \end{aligned}$$

where $\cap$ and $\cup$ respectively represent intersection and union of regions of two boxes, and $\left| \cdot \right|$ is the number of pixels in the region. The success plot shows the percentage of successful frames whose overlap ratio is beyond a pre-defined threshold varied from 0 to 1. The area under curve (AUC) score of the success plot is mainly adopted to rank the trackers.

### Quantitative evaluation with the light-weight trackers

#### Evaluation on DTB70

**Figure 5:**
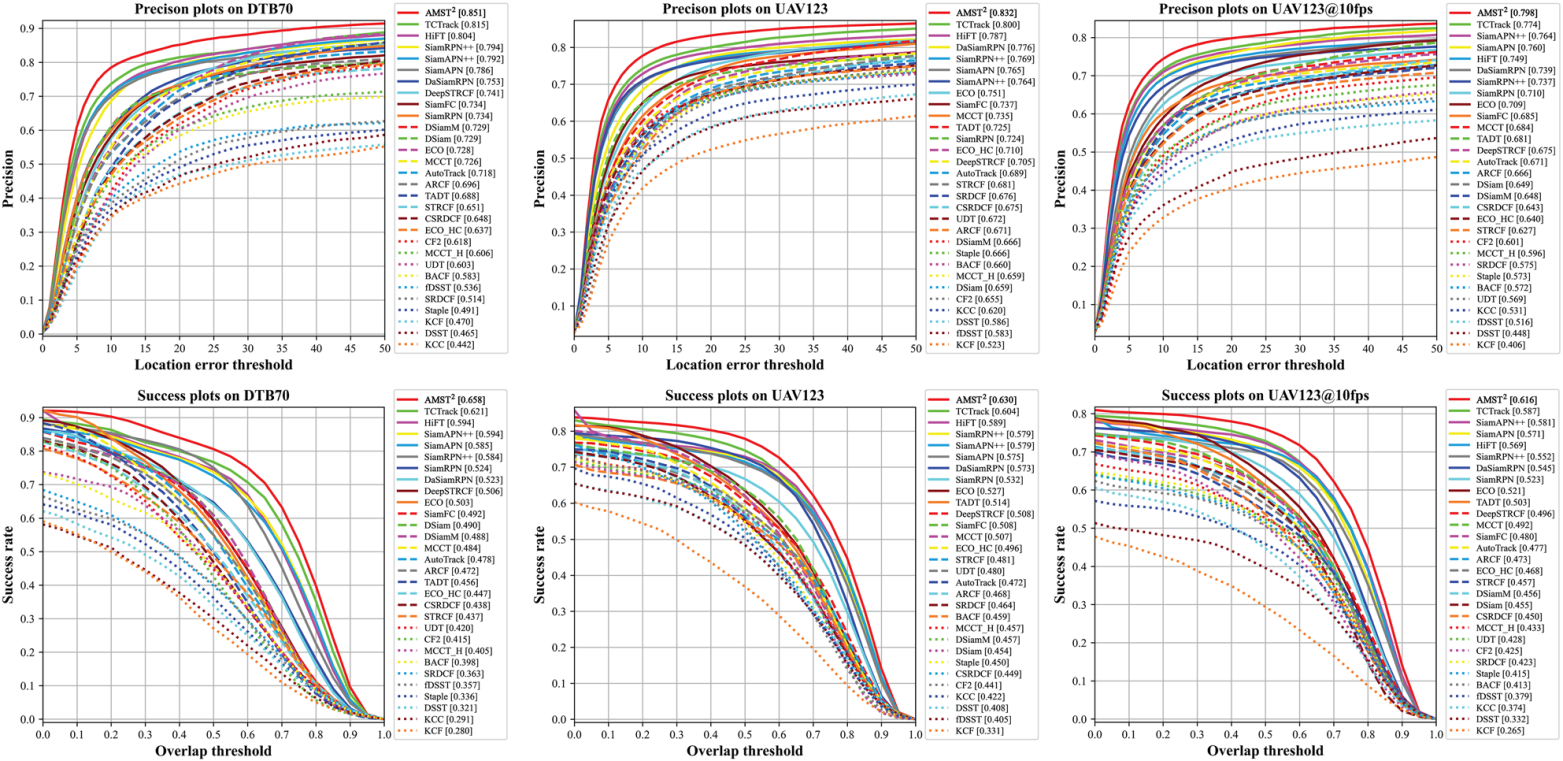
Comparison of overall performance with the light-weight trackers. The evaluation used the precision and success plots of the proposed tracker and 29 other light-weight trackers.

DTB70^68^ contains 70 challenging sequences constructed from data collected by UAVs. In addition, various challenging scenes with translation, rotation, and different size and aspect ratio due to camera motion further complicate the dataset. The robustness of our tracker in various complex scenarios caused by the fast motion of the UAV can be demonstrated with this benchmark. As a result of comparison with other trackers, AMST$\phantom{0}^2$ achieved precision (0.851) and success rate (0.658), ranking first place, and the results are shown in Fig. 5. Compared to the second-best and third-best place TCTrack (0.815) and HiFT (0.804), the precision improved by about 4.4$\%$ and 5.8$\%$, respectively. Similarly, in success rate, AMST$^2$ has 6.0$\%$ and 10.8$\%$ performance increase over TCTrack (0.621) and HiFT (0.594), respectively.

#### Evaluation on UAV123

The UAV123^69^ is a large-scale aerial tracking benchmark collected from an aerial viewpoint consisting of a total of 123 video sequences containing over 112 K frames. The object in the dataset are difficult to track due to large-scale change, illumination change, and occlusion, especially small object. As shown in Fig. 5, the AMST$\phantom{0}^2$ outperforms all other trackers for both precision and success rate. In terms of precision, the proposed method surpasses the second-best TCTrack (0.800) and third-best HiFT (0.787) by 4.0$\%$ and 5.7$\%$, respectively, with a precision score (0.832). The success rate also achieved an better performance increase of about 4.3$\%$ and 7.0$\%$, respectively, compared to the baseline trackers.

#### Evaluation on UAV123@10fps

The UAV123@10fps^69^ is downsampled by adopting the 10FPS image rate of the original version UAV123. The tracking problem is more challenge than the original version because the movement displacement and variation of the object are larger. As shown in Fig. 5, our tracker achieves the best performance in terms of both precision (0.798) and success rate (0.616). This clearly shows that our tracker is capable of robust tracking in discontinuous aerial data with no performance degradation due to image frame rate.

**Table 1:** Overall performance on UAV20L.

Trackers	Prec.	Succ.	Trackers	Prec.	Succ.
SRDCF	0.507	0.343	DaSiamRPN	0.677	0.519
BACF	0.584	0.415	TADT	0.609	0.459
DSiam	0.601	0.391	SiamRPN++	0.696	0.528
ECO	0.589	0.427	AutoTrack	0.512	0.349
STRCF	0.575	0.410	SiamAPN	0.717	0.532
DeepSTRCF	0.588	0.443	SiamAPN++	0.731	0.556
UDT	0.585	0.401	HiFT	**0.763**	**0.566**
ARCF	0.544	0.381	TCTrack	*0.780*	*0.580*
SiamFC	0.599	0.402	AMST$\phantom{0}^2$ (Ours)	***0.784***	***0.601***

The best three performances are respectively highlighted with bolditalic, italic, and bold.

#### Evaluation on UAV20L

The UAV20L^69^ was used for long-term tracking performance evaluation. This benchmark is a subset of UAV123 and consists of 20 long-term tracking sequences with an average of 2934 frames. As shown in Table 1, AMST$\phantom{0}^2$ attains first place with a precision of 0.784, ahead of second and third-best place TCTrack (0.780) and HiFT (0.763) by small margin of about 0.5$\%$ and 2.8$\%$, respectively. Also, the success rate of AMST$\phantom{0}^2$ has the best score (0.601), showing better tracking performance than TCTrack (0.580) and HiFT (0.566). This represents that the proposed method generates better features for tracking than existing methods on long-term datasets.

#### Evaluation on UAVTrack112$\_$L

UAVTrack112_L^70^ is a well-known long-term tracking dataset designed for aerial tracking, comprising of over 60,000 frames and a subset of UAVTrack112^70^. As demonstrated in Table 2, AMST$\phantom{0}^2$ is a more resilient tracker compared to state-of-the-art trackers. AMST$\phantom{0}^2$ secures the top spot with a precision score of 0.835, surpassing TCTrack (0.786) and SiamRPN++ (0.769) by approximately 6.2% and 8.6%, respectively. In terms of success rate (0.629), AMST$\phantom{0}^2$ also demonstrates superior performance to other trackers. These results confirm the superiority of our tracker over existing light-weight trackers in long-term benchmarks.

**Table 2:** Overall performance on UAVTrack112$\_$L.

Trackers	Prec.	Succ.	Trackers	Prec.	Succ.
SRDCF	0.508	0.320	DaSiamRPN	0.729	0.518
BACF	0.593	0.358	TADT	0.712	0.462
DSiam	0.643	0.400	SiamRPN++	**0.769**	0.557
ECO	0.684	0.436	AutoTrack	0.675	0.405
STRCF	0.609	0.360	SiamAPN	0.750	0.559
DeepSTRCF	0.713	0.460	SiamAPN++	0.741	0.546
UDT	0.620	0.388	HiFT	0.758	**0.570**
ARCF	0.640	0.400	TCTrack	*0.786*	*0.582*
SiamFC	0.690	0.452	AMST$\phantom{0}^2$(Ours)	***0.835***	***0.629***

The best three performances are respectively highlighted with bnolditalic, italic, and bold.

#### Attribute comparison

**Figure 6:**
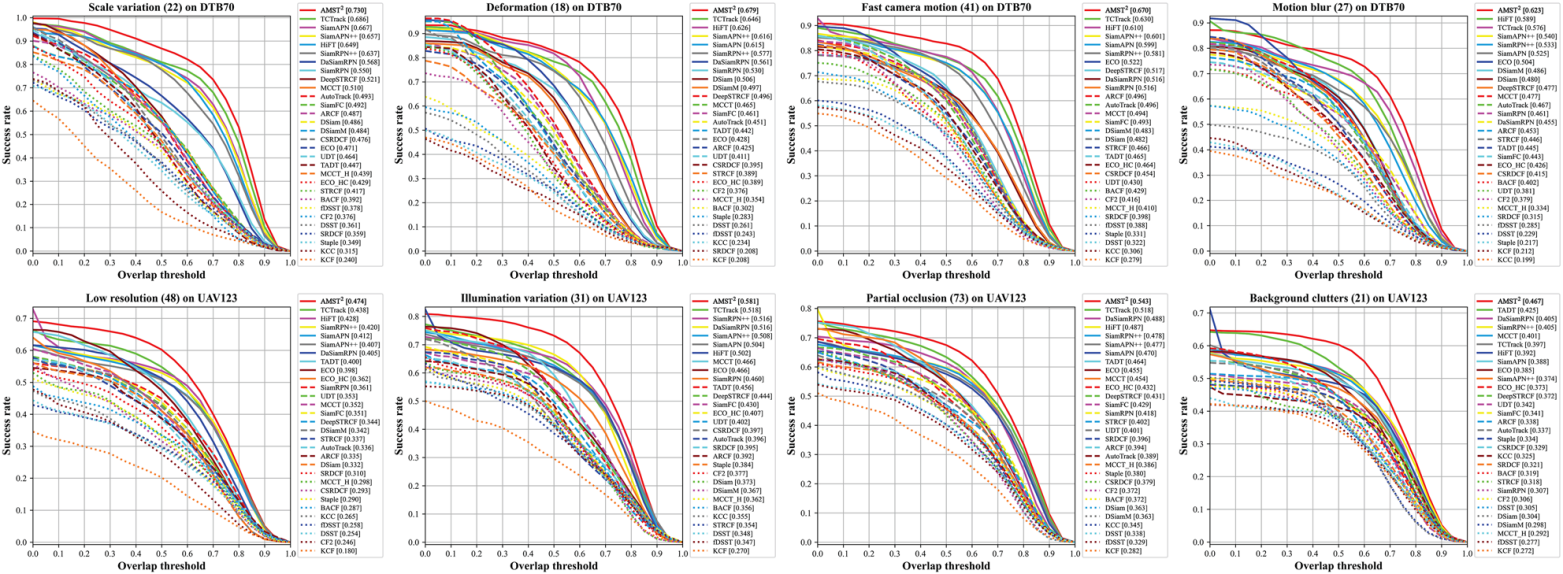
Success plots of OPE of the DTB70 and UAV123 dataset attributes. The several attribute-based evaluations on the DTB70 and UAV123 aerial tracking benchmarks.

**Figure 7:**
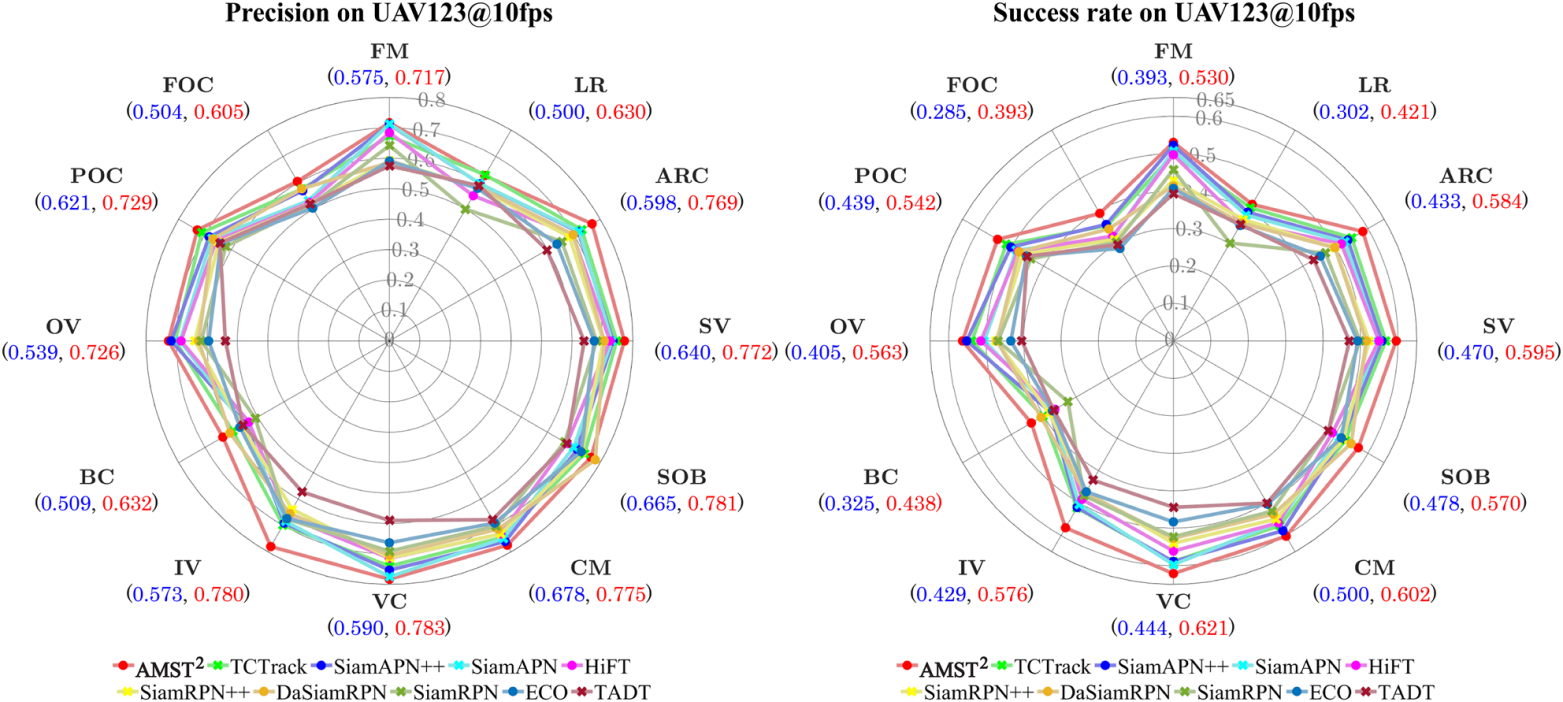
Overall performance of the UAV123@10fps dataset attributes. All of attribute-based evaluation of top 10 trackers on the UAV123@10fps aerial tracking benchmark. Red and blue fonts represent the highest and lowest scores, respectively.

Due to the severe motion of UAV, aerial tracking faces various challenges. Attributes were annotated in the benchmark datasets, as shown in Figs. 6 and 7 to evaluate the tracker performance under various challenging conditions.

Figure 6 illustrates that the proposed tracker outperforms other light-weight trackers in several challenging scenarios on the DTB70 and UAV123 benchmarks. Figure 7 depicts the evaluation results of all attributes on the UAV123@10fps benchmark. In terms of precision, our tracker secures the second-best position in low-resolution and similar object conditions, and first place in all other attributes. Particularly, AMST$\phantom{0}^2$ demonstrates the highest success rate among all attributes in the UAV123@10fps dataset. By utilizing multi-level spatial and temporal information, our tracker exhibits exceptional performance in various scenarios, such as scale variation, deformation, fast camera motion, and occlusion, among others. Moreover, template updates at the template feature level provide an advantage of more robust tracking for extreme variations.

#### Ablation study

**Table 3:**
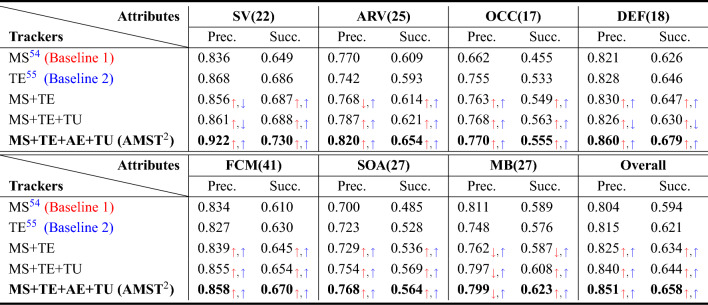
Ablation analysis on DTB70 dataset.

The red and blue arrows denote improvement compared to baseline 1 and baseline 2, respectively and the down and up arrows indicate scores lower and higher than baseline, respectively.

To validate the impacts of the proposed method, we performed several ablation studies on DTB70 dataset. We evaluated five variants of our tracker, including: (1) MS, which uses only the features of the multi-level spatial encoder as the first baseline, (2) TE, which utilizes only a temporal encoder as the second baseline, (3) MS+TE, which applies both multi-level spatial and temporal encoders, (4) MS+TE+TU, a model in which a template update network is added to MS+TE, and (5) MS+TE+AE+TU, the final model that includes the aggregation encoder added to MS+TE+TU. In this ablation study, the same multi-context decoder structure was used about the method of applying both multi-level spatial and temporal information. As shown in Table 3, our contribution not only demonstrates outstanding performance in various complex conditions, but also shows the highest score in precision and success rate.

### Quantitative evaluation with the deep trackers

**Figure 8:**
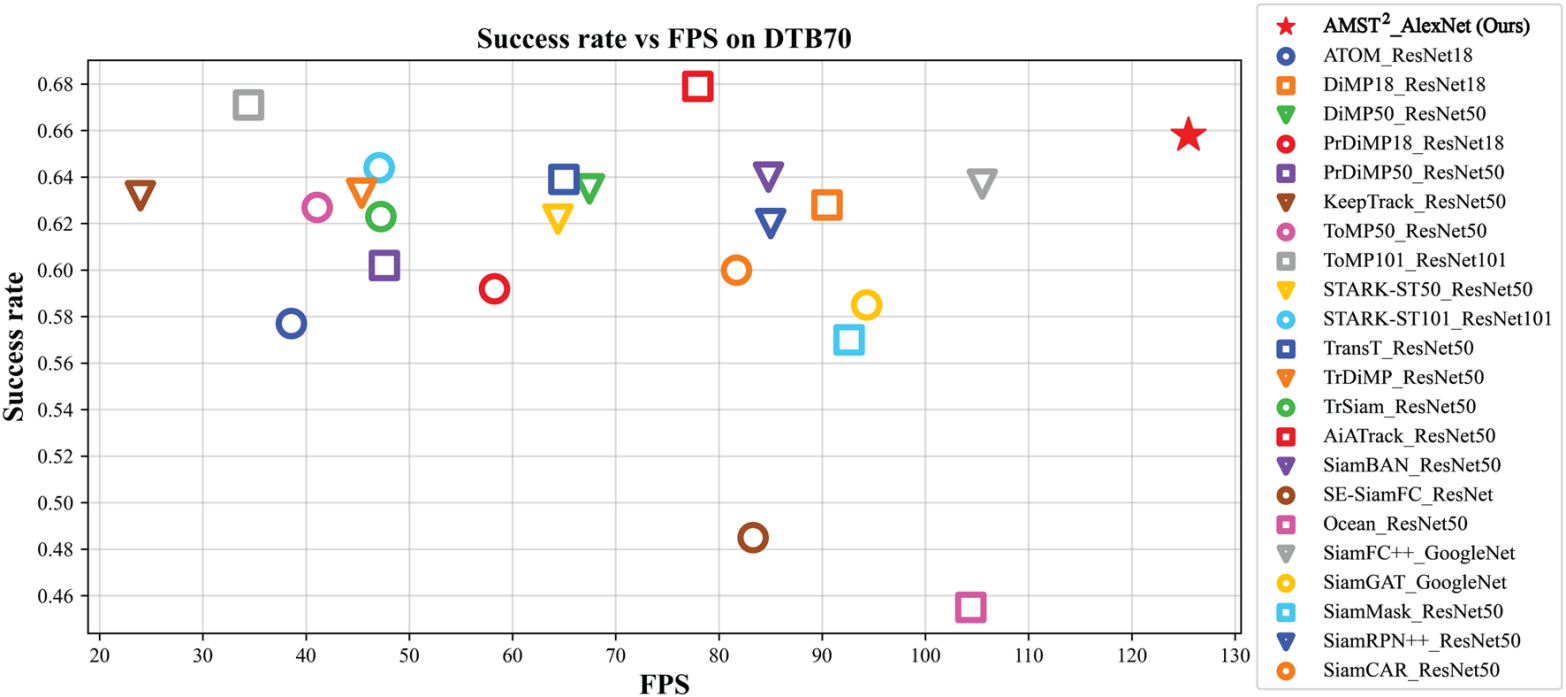
The comparison of the quality and speed of state-of-the-art trackers with deeper backbones on DTB70. The trackers used for comparison consist of trackers that adopt a deeper backbone network than AlextNet.

**Figure 9:**
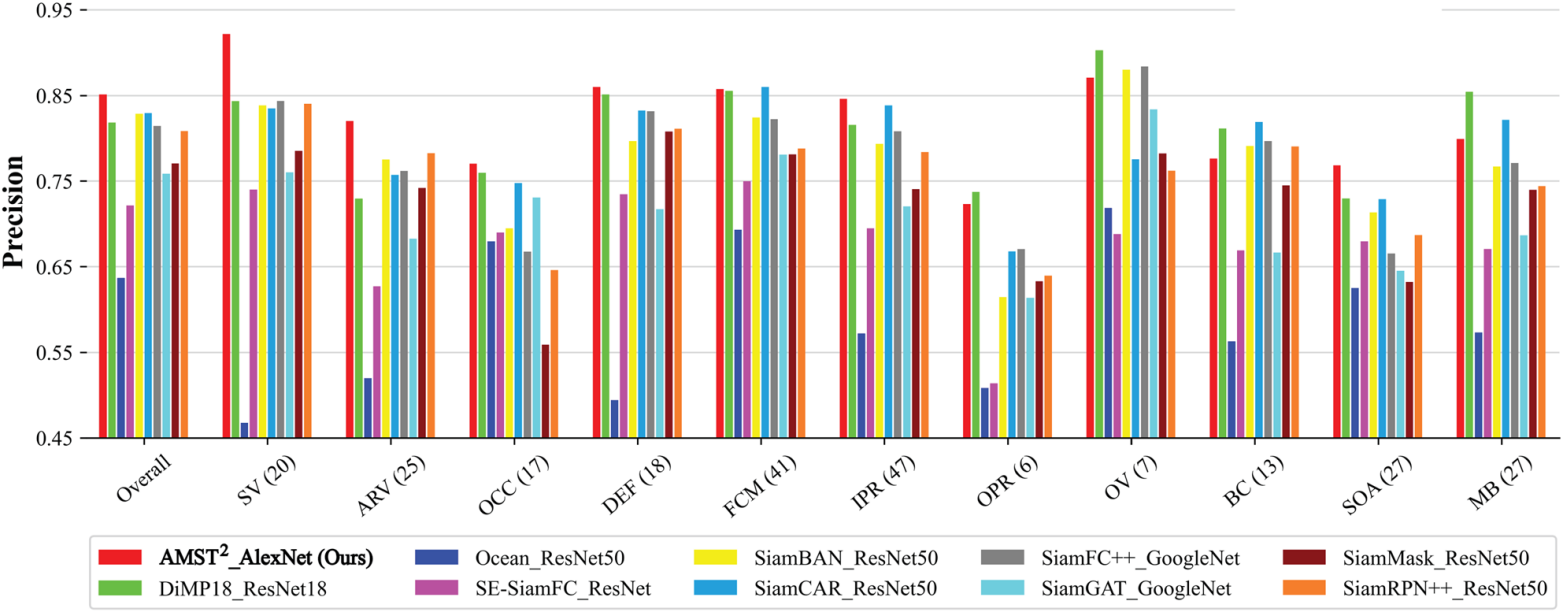
Attribute-based comparison results of trackers with deeper backbones. The trackers used for comparison are composed of trackers with the top 10 running speed among deep trackers.

**Table 4:** Comparisons to trackers with deeper backbones and baselines on VisDrone-SOT2020 and UAVDT.

Benchmark	Param (M)	Avg. FPS.	VisDrone-SOT2020		UAVDT	
			Prec.	Succ.	Prec.	Succ.
Trackers						
SiamBAN$\_$ResNet50	53.9	86.1	0.797	0.585	0.806	0.601
SiamGAT$\_$GoogleNet	14.2	94.8	0.811	0.607	0.754	0.574
SiamMask$\_$ResNet50	21.5	92.6	0.806	0.588	0.782	0.580
SiamRPN++$\_$ResNet50	54.0	86.3	0.778	0.592	0.801	0.594
SiamCAR$\_$ResNet50	51.4	84.7	0.838	0.630	0.804	0.598
DiMP18$\_$ResNet18	19.7	89.2	0.784	0.592	0.774	0.567
DiMP50$\_$ResNet50	43.1	76.1	0.819	0.621	0.798	0.594
PrDiMP18$\_$ResNet18	19.7	60.8	0.777	0.588	0.767	0.577
PrDiMP50$\_$ResNet50	43.1	50.2	0.806	0.605	0.830	**0.618**
KeepTrack$\_$ResNet50	41.7	24.1	0.844	0.638	0.825	0.610
ToMP50$\_$ResNet50	46.4	40.6	0.840	**0.639**	*0.853*	***0.644***
ToMP101$\_$ResNet101	65.4	35.1	**0.845**	*0.643*	0.808	0.612
STARK-ST50$\_$ResNet50	28.2	53.0	0.735	0.582	0.740	0.551
STARK-ST101$\_$ResNet101	47.2	37.9	0.755	0.587	0.704	0.523
TrDiMP$\_$ResNet50	42.3	42.6	0.830	0.627	***0.860***	*0.633*
TransT$\_$ResNet50	23.0	64.8	***0.868***	***0.653***	0.832	0.612
HiFT$\_$AlexNet	10.4	159.7	0.784	0.570	0.734	0.522
TCTrack$\_$AlexNet	9.8	146.1	0.828	0.604	0.773	0.570
AMST$\phantom{0}^2$ $\_$AlexNet (Ours)	11.6	122.3	*0.863*	0.628	**0.835**	0.614

The best three performances are respectively highlighted with bolditalic, italic and bold. In addition, inference time and parameters are further compared to prove the superiority of the proposed tracker in the aerial datasets.

Our goal was to enhance the robustness of our proposed aerial tracking by combining multi-level spatial and temporal information, and thus handle complex conditions. To obtain clearer results, we compared our method with 22 state-of-the-art trackers with deeper backbones. As depicted in Fig. 8, even though our method uses a light-weight backbone, it achieves competitive performance with a significantly faster tracking speed than AiATrack, which has the highest success rate. Furthermore, we conducted comparison experiments on all scenarios of the DTB70 using the top 10 tracking speed-based trackers to support the attribute-based analysis with deep trackers. As shown in Fig. 9, our tracker outperforms others in various complex and cluttered scenarios. The proposed robust feature representation, which aggregates multi-level spatial and temporal context, reduces the performance gap with deeper backbone-based trackers and ensures efficient and robust tracking in various aerial scenes. Table  4 presents an in-depth comparison between the proposed method and deeper backbone-based trackers, as well as baseline trackers. we conducted evaluations on multiple factors including frames per second (fps), parameters, and performance metrics using well-known aerial datasets such as VisDrone-SOT2020^71^ and UAVDT^72^. VisDrone-SOT2020 is based on data collected from numerous real-world situations on weather and lighting variations, and UAVDT also includes various frames in complex scenarios that confuse tracker performance such as weather, altitude, camera view, object appearance, and occlusion. For clarity, STARK and TransT use a modified version of ResNet that removes the last stage, so they have a fewer number of parameters than trackers using the other deeper backbones. HiFT, TCTrack, and the proposed tracker show faster processing time with much less parameters and tracking speeds of more than 100 fps than deep trackers. In addition, HiFT and TCTrack have advantages in parameters and fps over the proposed tracker, but in terms of performance, they underperform deep trackers and the proposed tracker. Furthermore, our proposed tracker not only demonstrates lower parameter complexity compared to TransT, which achieved the highest score in VisDrone-SOT2020, but also exhibits similar precision performance and comparable success performance to deeper backbone models, even with a doubled fps. These results highlight the efficiency and effectiveness of our proposed tracker in terms of parameter usage and overall tracking performance, showcasing its potential for real-time aerial tracking applications. In the UAVDT dataset, the proposed method shows a comparable performance to state-of-the-art trackers, while maintaining low parameter complexity and fast processing speed. These findings further demonstrate the effectiveness and efficiency of our proposed method in aerial tracking tasks. Among the deeper backbone-based trackers, there are trackers close to 100 fps, but the proposed tracker outperforms in terms of parameters and performance. Therefore, our tracker demonstrates higher efficiency in aerial tracking using UAVs than many SOTA trackers with low latency, fast tracking speed and superior performance.

## Conclusion

In this paper, we presented the aggregated multi-level spatial and temporal context-based transformer (AMST$\phantom{0}^2$) architecture, a novel approach for robust aerial tracking that leverages multi-level spatial and temporal information through a Transformer-based model. The proposed approach includes an aggregation encoder that enhances the similarity map and a multi-context decoder that generates powerful refined similarity maps. The utilization of an aggregated multi-level spatial and temporal information-based transformer, along with a light-weight backbone, effectively addresses the challenges of tracking speed and aerial tracking when employing UAVs. The adoption of a template update process further enhances the robustness of our approach against complex scenarios.

Extensive experiments on challenging aerial benchmarks, including DTB70, UAV123, UAV123@10fps, UAV20L, and UAVTrack112$\_$L, demonstrated that AMST$\phantom{0}^2$ outperforms state-of-the-art methods in terms of both accuracy and efficiency.

While our approach shows promising results, there are still limitations to be addressed, such as the sensitivity to low-lighting conditions and the need for a large amount of training data. Future research can investigate ways to overcome these limitations and further improve the accuracy and efficiency of aerial tracking. Overall, the proposed approach represents a significant advancement in the development of more robust and effective aerial tracking systems.

## Data Availability

All data generated or analyzed in this study are included in this published article. The training and testing datasets used in this study are publicly available and have been cited in accordance with research rules. Detailed descriptions of the datasets and their citations can be found in the “Experimental results” section of the paper. For instance, the ImageNet VID dataset’s training set can be downloaded from the link https://image-net.org/challenges/LSVRC/2015/index.php. The COCO dataset’s training set can be downloaded from https://cocodataset.org/#home, while the GOT-10K dataset’s training set can be downloaded from http://got-10k.aitestunion.com/. Furthermore, the LaSOT dataset’s training set can be accessed via http://vision.cs.stonybrook.edu/~lasot/. The testing sets of the DTB70 dataset, the UAV123, UAV123@10fps and UAV20L datasets, and the UAVTrack112_L dataset, VisDrone-SOT2020 dataset and UAVDT dataset can be downloaded from https://github.com/flyers/drone-tracking, https://cemse.kaust.edu.sa/ivul/uav123, https://github.com/vision4robotics/SiamAPN, http://aiskyeye.com/, and https://sites.google.com/view/grli-uavdt, respectively.
