# Correction: AMST^2^: aggregated multi-level spatial and temporal context-based transformer for robust aerial tracking

**DOI:** 10.1038/s41598-025-00188-y

**Published:** 2025-05-26

**Authors:** Hasil Park, Injae Lee, Dasol Jeong, Joonki Paik

**Affiliations:** 1 Department of Image, Chung-Ang University, 84 Heukseok-ro, Seoul, 06974 Korea; 2 Department of Artificial Intelligence, Chung-Ang University, 84 Heukseok-ro, Seoul, 06974 Korea

Correction to: *Scientifc Reports* 10.1038/s41598-023-36131-2, published online 04 June 2023

The original PDF version of this Article contained errors in Equations 2, 4, 5, 6, 7, 8, 9, 10, 11, 12, 13, 14, 15 and 16, where mathematical expressions were partially not italicized. Additionally, Equation 17 contained errors, where a tilde was omitted, and mathematical expressions were partially not italicized.

These errors have now been corrected in the PDF version of the Article; the HTML version was correct from the time of publication.

Furthermore, Figure 3 contained an error in panel (**a**), where an arrow pointing to “Add & Norm” was omitted. The original Figure 3 and accompanying legend appear below.

**Fig. 3:**
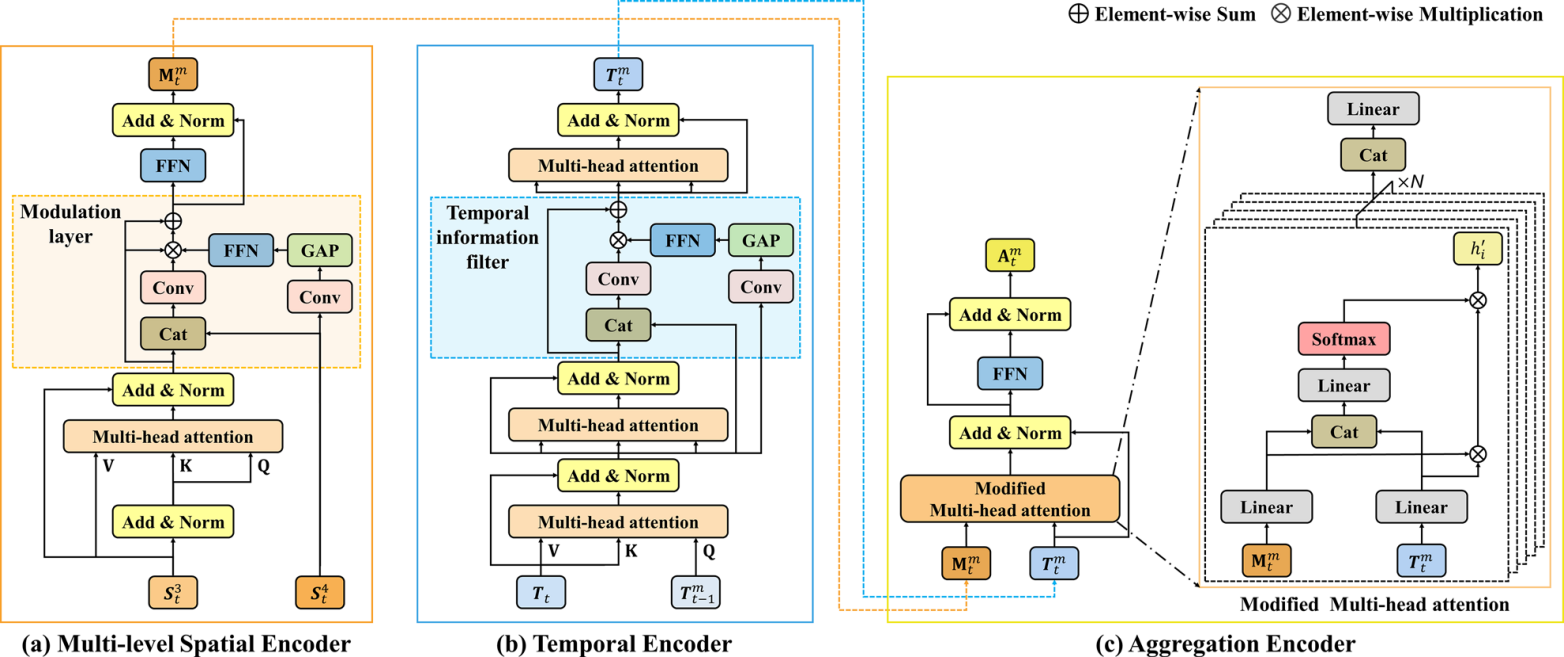
Architecture of the proposed transformer encoder. The proposed encoder consists of three components: a multi-level spatial encoder, a temporal encoder, and an aggregation encoder.

The original Article has been corrected.

